# Findings of Esophagogastroduodenoscopy in Patients Suspected of Upper Gastrointestinal Bleeding Referred to the Main Endoscopy Unit at King Fahad Specialist Hospital

**DOI:** 10.7759/cureus.11862

**Published:** 2020-12-03

**Authors:** Abdallah Alatawi, Wejdan S Aljohani, Rabab T Aljayani, Yassmeen Alblowi, Maisaa Yousuf, Hadeel Almutairi

**Affiliations:** 1 Internal Medicine Department, University of Tabuk, Tabuk, SAU

**Keywords:** upper gastrointestinal bleeding, endoscopy, duodenal ulcer, gastric ulcer, variceal causes, saudi arabia

## Abstract

Introduction

Upper gastrointestinal bleeding (UGIB) is defined as any blood loss originating from the esophagus, stomach or the proximal duodenum above the ligament of Treitz. Ethnic trends regarding the causes of UGIB have been reported. The aim of this study was to identify the most common causes of UGIB among patients residing in Tabuk city, Saudi Arabia.

Methods

We have conducted a retrospective descriptive cohort study at King Fahad Specialist Hospital (KFSH), Tabuk, Saudi Arabia. All adult patients above the age of 18 years who were suspected of UGIB and referred for esophagogastroduodenoscopy (EGD) were included. The collected data included age, sex, nationality, complaint, EGD, and histopathologic findings.

Results

Between January 1, 2017 and December 31, 2019, 73 patients were included. 83.6% were Saudi, and 64.4% were males. Hematemesis was the main complaint (65.8%). Esophagogastroduodenoscopy was normal in 6.84% of cases; however, it showed UGIB due to esophageal and gastric varices (9.57%) as well as non-variceal causes (83.56%). The most frequent non-variceal findings which represent about two-thirds of the cases were duodenal ulcer (20.53%), antral gastropathy (13.68%), gastric ulcer (12.32%), antral gastritis (10.94%), and duodenal/gastric mass (9.57%), whereas much less frequent findings representing a total of 16.39% of cases included Cameron gastropathy, gastropathy/duodenopathy, esophagitis/gastritis, gastritis/duodenitis, gastroesophageal reflux disease (GERD), and Mallory-Weiss tear.

Conclusion

Non-variceal causes showed higher prevalence as causes of UGIB than variceal causes in the Tabuk area. Furthermore, chronic duodenal and gastric ulcers were the most common culprits of bleeding, whereas duodenitis, gastritis, esophagitis, and Mallory-Weiss syndrome were the least common non-variceal causes.

## Introduction

Upper gastrointestinal bleeding (UGIB) is defined as any blood loss originating from the esophagus, stomach or the proximal duodenum above the ligament of Treitz. It is a life-threatening event commonly encountered in the emergency and hospital setting [1]. Mortality varies (6-14%), and it depends on the degree of initial blood loss, the rate of rebleeding after endoscopy, the underlying disease and the patient's age [2]. 

Upper gastrointestinal bleeding can manifest as hematemesis and/or melena. Hematochezia is occasionally seen in 5-10% of patients having a massive UGIB source. Patients can also present with symptoms secondary to blood loss, such as pallor, tachycardia, syncopal episodes, fatigue, and weakness [3].

The causes of UGIB can be variceal or non-variceal [4]. Esophageal and/or gastric varices are related to portal hypertension and cirrhotic liver disease. Globally, peptic ulcer disease accounts for 40% to 50% of the non-variceal causes. *Helicobacter pylori* infection, the use of nonsteroidal anti-inflammatory or low dose aspirin, and stress-related mucosal disease are the most frequent causes [5]. Aside from peptic ulcer disease, erosive esophagitis, duodenitis, Mallory-Weiss tear, and vascular malformations constitute other factors, which could precipitate UGIB [6].

Esophagogastroduodenoscopy (EGD) is the mainstay treatment in all patients suspected of having UGIB to identify the cause and potentially treat the source of bleeding. Following resuscitation and stabilization of hemodynamic parameters by proton pump inhibitors, vasopressors, intravenous fluids, and blood transfusion, EGD should be done within 24 hours of admission. However, endoscopy should be done earlier within 12 hours in high-risk patients with bloody nasogastric aspirate associated with tachycardia and hypotension [7].

Ethnic trends regarding the causes of UGIB has been reported. Hispanics were likely to have esophageal varices, whereas blacks were likely to have ulcers. Whites represented both ulcers and varices equally. As well, the risk and severity of UGIB are highly related to the population's lifestyles [8].

Due to a paucity of data in our region, this study was designed to identify the commonest causes of UGIB among patients residing in Tabuk, Saudi Arabia.

## Materials and methods

Ethical considerations

The study obtained ethical approval from the Research Ethics Committee of the Faculty of Medicine, University of Tabuk, Tabuk, Saudi Arabia (Number: READ 0107, Date: 11/08/2020). Informed consent was waived. To ensure privacy, confidentiality, and anonymity, each participant was assigned a numeric code on his respective data collection sheet. No identifying information is included in the article.

Study design and setting

This retrospective cohort study was conducted between January 1, 2017 and December 31, 2019 at King Fahad Specialist Hospital (KFSH), Tabuk, Saudi Arabia.

All upper endoscopy reports during this period were reviewed. We collected the data of all patients who were suspected of UGIB and had EGD procedure. The histopathology reports were assessed, and the causes and most common findings were identified.

Study population

The target population included all adult patients admitted to KFSH for whom an EGD was requested in suspicion of UGIB at the main hospital of Tabuk region, Saudi Arabia. It is a region with a population of around 900,000 and is served by many peripheral and two main hospitals.

Inclusion criteria

We included all male and female adult (above 18 years old) patients living in the Tabuk region, Saudi Arabia who were suspected of UGIB and had EGD procedure at KFSH between January 1, 2017 and December 31, 2019.

Sample size

Considering a population size of 910,030, a confidence level of 95%, and a margin of error of 5%, we had a sample size of 384. This means that 384 or more measurements/surveys are needed to have a confidence level of 95% that the real value is within ±5% of the measured/surveyed value.

Sampling technique

Stratified random probability sampling was used.

Study variables

Age, sex, medication history, associated disease, level of education, and compliance with treatment were the study variables.

Data collection tools

Patients’ electronic files and medical records containing endoscopy reports were collected as scientific tools to be reviewed in a spreadsheet XL Windows Microsoft Office 360. A review of the literature was conducted using PubMed research engine and the Saudi digital library.

Statistical analysis

Data were analyzed using the Statistical Package for the Social Sciences, version 22 (SPSS; IBM Corp., Armonk, NY, USA). Categorical variables were presented as the frequency and percentage, while continuous variables were presented as median and interquartile range.

## Results

During the study period a total of 327 patients were referred to the main endoscopy unit at King Fahad Specialist Hospital for EGD procedure, among them 73 patients were suspected of UGIB. The majority (83.6%) were Saudi, and about two-thirds (64.4%) of them were males. Their age ranged from 18.0 to 67.0 with a median of 45.0 (interquartile range (IQR)=21.0-49.0) years. Hematemesis was the main complaint (65.8%), while melena represented about one-fourth (28.8%) as shown in Table 1.

**Table 1:** Demographic data of the studied patients (N=73). IQR: interquartile range

Age (years)	Minimum		18.0
	Maximum		67.0
	Median		45.0
	IQR		21.0-49.0
Sex	Female	N	26
		%	35.6%
	Male	N	47
		%	64.4%
Nationality	Saudi	N	61
		%	83.6%
	Non-Saudi	N	12
		%	16.4%
Complaint	Hematemesis	N	48
		%	65.8%
	Melena	N	21
		%	28.8%
	Hematemesis and melena	N	4
		%	5.5%

**Figure 1:**
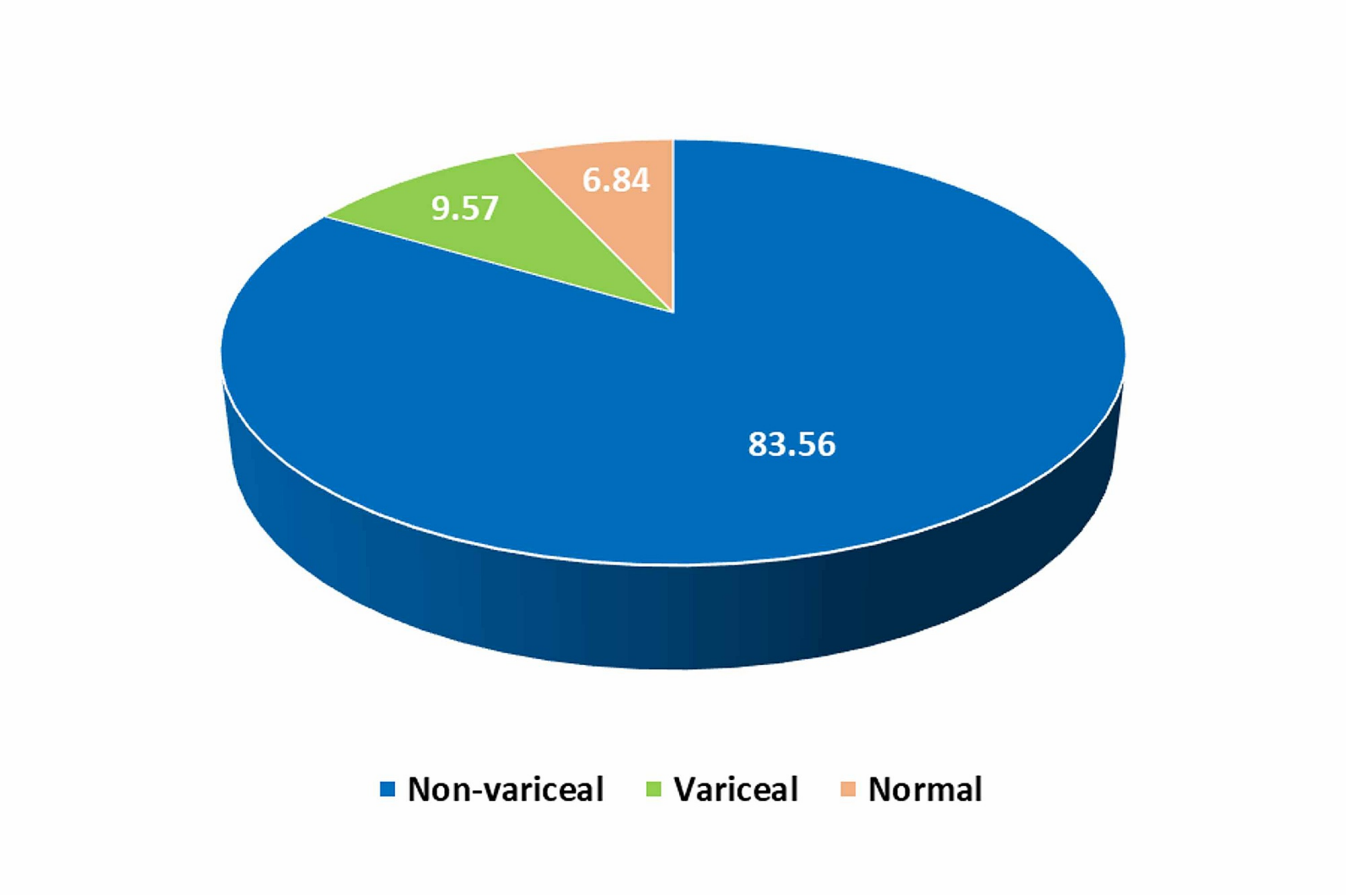
The main esophagogastroduodenoscopy findings in the studied patients.

Figure 1 illustrates the main EGD findings in the studied patients. The most common causes of UGIB were non-variceal (83.56%), while UGIB due to esophageal or gastric varices represented 9.57%.

The most frequent non-variceal findings were chronic duodenal and gastric ulcers (20.53% and 12.23%, respectively). Gastric or duodenal masses were found in seven patients (9.57%). There were two patients (2.73%) who showed gastroesophageal reflux disease (GERD) complicated by Mallory-Weiss tear. In about one-fourth (24.62%) of the studied patients, no cause of bleeding was identified and the EGD showed antral gastropathy (13.68%) and antral gastritis (10.94%). Variceal causes of UGIB included either esophageal varices alone (6.84%) or combined esophageal and gastric varices (2.73%) (Table 2).

**Table 2:** The frequency of esophagogastroduodenoscopy findings in the studied patients (N=73). GERD: gastroesophageal reflux disease; EGD: esophagogastroduodenoscopy

		N=73	%
Findings	Non-variceal findings		
	Duodenal ulcer	15	20.53%
	Antral gastropathy	10	13.68%
	Gastric ulcer	9	12.32%
	Antral gastritis	8	10.94%
	Duodenal/gastric mass	7	9.57%
	Cameron gastropathy	3	4.1%
	Gastropathy and duodenopathy	3	4.1%
	Esophagitis + gastritis	2	2.73%
	Gastritis + duodenitis	2	2.73%
	GERD and Mallory Weiss tear	2	2.73%
	Variceal		
	Esophageal varices	5	6.84%
	Esophageal and gastric varices	2	2.73%
	Normal EGD	5	6.84%

Reports of histopathologic findings of endoscopy-obtained biopsies were available in 17 patients. Mild to moderate chronic nonspecific gastritis were the most frequent findings (52.94%). Additionally, *Helicobacter pylori* bacilli were detected in eight (47.05%) patients (Table 3).

**Table 3:** Histopathologic findings in the studied patients (N=17).

	N=17	%
Histopathologic findings		
Mild to moderate chronic nonspecific gastritis	9	52.94
Moderate chronic active gastritis	2	11.76
Severe chronic active gastritis	1	5.88
Mild chronic nonspecific duodenitis	2	11.76
Moderate chronic nonspecific duodenitis	2	11.76
Very mild esophagitis	1	5.88
Helicobacter pylori bacilli	8	47.05

## Discussion

This study revealed that non-variceal causes were more common than variceal ones. The most common non-variceal etiologies were chronic duodenal and gastric ulcers, followed by duodenal or gastric masses, duodenitis, gastritis, esophagitis, and Mallory-Weiss syndrome.

In this study, the median age of the studied patients was 45 years, and males outnumbered females (approximately 2:1 ratio). This finding coincides with several reports obtained from a prospective study on patients with UGIB in Iceland [9], a multicenter study in USA [8], a cohort of adult Sri Lankans [10], and a tertiary care center in Nepal [11].

Hematemesis was the main presentation (65.8%) of UGIB in this study, while melena was less frequent. Similarly, Kim et al. reported higher frequency of hematemesis than melena and hematochezia among patients with UGIB who presented to Gastrointestinal and Liver Diseases Division, University of Southern California, Los Angeles [12].

The present study explored the most common causes of UGIB being non-variceal (83.56%) as well as esophageal or gastric varices (9.57%). Various studies reported similar higher frequency of nonvariceal than variceal causes. Kim et al. showed that ulcers were more common than varices and erosive esophagitis [12]. Approximately one-fourth (22.78%) of our patients exhibited chronic duodenal and gastric ulcers. However, duodenal and gastric ulcers accounted for 43% of patients with UGIB in Nepal, followed by variceal bleeding in 23% of cases [11]. Another study in Sri Lanka identified severe erosive antral gastritis and duodenitis as the most frequent causes of UGIB, while chronic duodenal and gastric ulcers were less frequent [10]. The predominance of non-variceal causes has also been stated in several studies from different geographical areas [13,14].

Another study included 404 Saudi patients complaining of dyspepsia reported a high (46.5%) prevalence of *H. pylori* as confirmed by endoscopically obtained gastric biopsies [15]. This might contribute to the high frequency of peptic ulcer disease in this study. This has also been proven in histopathological findings of biopsies that were taken during endoscopy. Although a decline in the incidence of duodenal ulcers has been suggested, adaptation of the population to modern lifestyle and unhealthy food practices constitute a high risk that might precipitate upper GIT bleeding. Moreover, an interaction between dietary habits and the virulence of *H. pylori* infection has recently been proven [16,17].

The observed high frequency of acute gastric and duodenal inflammation among Saudi subjects might be attributed to poor knowledge about the risk of improper use of nonsteroidal anti-inflammatory drugs. Heavy use of these drugs has been linked to gastritis and GERD [18].

Alternatively, analysis of patients with UGIB and liver disease revealed that variceal bleeding, either esophageal or gastric, was responsible for most of the UGIB attacks (57.7%) [19-21]. It has been reported that about half of cirrhotic patients will develop varices due to portal hypertension and 40% of them will have variceal bleeding [22]. Therefore, routine EGD is recommended for all cirrhotic patients [23].

Analysis of etiological trends of UGIB is very important as each cause of bleeding carries different epidemiologic features and outcomes including death rates. Variceal bleeding has been linked to higher mortality rates compared to peptic ulcer disease [24]. However, innovations in endoscopic techniques for bleeding management have contributed to the decline in death rates [25]. This carries important implications regarding the future prevention and management measures as well as future research.

This study has some limitations including its retrospective design, being carried out in a single tertiary care hospital, in addition to the small sample size and the lack of accurate classifications of ulcers in the EGD reports.

## Conclusions

This study explored the current trends in the etiologies of upper GIT bleeding among patients referred to the main endoscopy unit at King Fahad Specialist Hospital. It shows higher prevalence of non-variceal causes than variceal ones.

Furthermore, chronic duodenal and gastric ulcers were the most common culprits of bleeding, followed by duodenal or gastric masses, duodenitis, gastritis, esophagitis, and Mallory-Weiss syndrome.
